# Investigation and Modelling of the Weight Wear of Friction Pads of a Railway Disc Brake

**DOI:** 10.3390/ma15186312

**Published:** 2022-09-12

**Authors:** Wojciech Sawczuk, Agnieszka Merkisz-Guranowska, Dariusz Ulbrich, Jakub Kowalczyk, Armando-Miguel Rilo Cañás

**Affiliations:** 1 Faculty of Civil and Transport Engineering, Institute of Transport, Poznan University of Technology, 60-965 Poznan, Poland; 2 Faculty of Civil and Transport Engineering, Institute of Machines and Motor Vehicles, Poznan University of Technology, 60-965 Poznan, Poland; 3 Doctoral School of Poznan University of Technology, Poznan University of Technology, 60-965 Poznan, Poland

**Keywords:** pad wear, weight wear, bench testing, railway disc brake

## Abstract

This paper presents the results of tests on the railway disc brake with regard to the weight wear of friction pads. The tests were carried out at a certified brake test bench where the friction-mechanical characteristics of the railway brake were determined. The test stand was additionally equipped with a thermal imaging camera to observe the contact between the brake pads and the brake disc. The scientific goal of the test is to evaluate the relationship between the weight wear of friction pads and the quantities characterizing the braking process. The quantities characterizing the braking process included pad-to-disc contact area, friction pad thickness, pad-to-disc pressure, and braking speed. A regression model to estimate the friction pad wear on the basis of a single braking with the given input quantities was determined. The greatest influence on the increase in weight wear of friction pads has the braking velocity, which was confirmed by the value of the correlation coefficient of the regression model at value 0.81. The pressure of the friction pad to the disc and the friction pad thickness do not have a significant effect on the weight wear described by the regression model, and the obtained correlation coefficient for these parameters was lower than the value of 0.2.

## 1. Introduction

Road transport is one of the main human activities that affects the emission of harmful substances to the environment. For this reason, more and more stringent European emission standards are being introduced [1]. These restrictions mainly concern limiting combustion emissions coming from internal combustion engines, both gasoline and diesel [2,3]. Another important source of pollution from motor vehicles is the chassis system, specifically the wear products from tires [4,5]. For several years, environmentalists have been pointing out that in addition to the emission of harmful substances from internal combustion engines, there is also the emission in the braking systems. Vehicle friction brakes are the cause of metallic and non-metallic friction products and the emission of toxic gases to the environment. A separate and important issue is the pollution of the environment by worn out tires from motor vehicles. Studies by Glišović et al. [6] have proven that road as well as rail vehicles using friction brakes pollute the area around roads and railroad tracks. This is mainly dust in the form of particulate matter. This has the effect of polluting the environment and deteriorating the health of the occupants near roads and railroads. For humans and animals, the most harmful elements formed during braking are copper and antimony powders. Particulate matter (PM) emissions from the friction brakes depend on the physicochemical properties of the friction material and on the amount of braking and its intensity.

In friction brakes for rail vehicles attention of designers, constructors and researchers are focused on ensuring friction characteristics at a level compliant with requirements in UIC or EN-PN regulations [7,8]. Due to the operation of the brakes, brake performance must be maintained in all weather conditions (heavy rain, snow, or dirt on the rails and road) without extending the braking distance. Research on friction brakes is carried out for a long time until a compromise is reached, i.e., good friction characteristics with acceptable friction material wear and manufacturing costs. This is why friction pad manufacturers select such a composition of materials for friction pads in their laboratories to achieve a compromise between the best friction-mechanical characteristics and acceptable wear. For the last few years, environmental issues have also started to concern manufacturers of brake friction pads. In the 1970s, it was found that asbestos, which is the main component of friction brakes, has carcinogenic properties for humans and must absolutely be removed from brake pads and friction pads [9,10]. This material is not used for friction pads, although, as a material combined with copper fibers, it is the best material for brakes due to its resistance in high temperatures and stability of brake friction characteristics [11,12]. Therefore, new materials are being sought to replace asbestos while maintaining the required brake friction characteristics [13]. Research has been carried out on the effect of asbestos-free particle size within 125–710 µm on the wear of a friction material in brake pads [14]. The issue of emission of wear products from the braking system to the environment has been addressed in many articles [6,15]. Attention has been paid to issues related to environmental pollution and the impact of wear products on the health of people in the neighborhood of roads [16], in particular particles in the air [17,18] and fine particulate matter falling to the ground, which are toxic because animals and humans inhale them into the body [19,20]. Regardless of the dustiness of the environment as a result of friction brakes, another adverse environmental issue is friction material waste. This is the metal plate with the remaining unused part of the friction material [21]. Therefore, research has also been undertaken to reduce the proportion or completely replace copper with other components with similar properties [19,22]. Therefore, many scientific laboratories around the world have begun to carry out research work on replacing toxic and dangerous to humans and animals components of friction pads with new ones, which are based on different types of phenolic and epoxy resins [14], the addition of carbon fibers and basalt [23], fibers with a low content of copper, e.g., 7% [24,25,26], fibers with palm grain and others [16], asbestos-free sugarcane ashes as fillers [27], or such components as titanium [28,29]. Some studies have been conducted to use fruit peel waste, such as banana, instead of typical phenolic or phenol-formaldehyde resins and asbestos [30]. These studies in friction characteristics of friction materials have shown that banana fruit peel waste can be a good substitute for toxic and carcinogenic brake pad elements. Additionally, friction pad wear issues were tested on steel discs with various alloying additives [31]. In the field of new friction materials, tribological research is carried out to determine frictional–mechanical characteristics to check the possibility of using pads in the long operation of brake pads until complete wear, as in snowfall and rain [32] without adverse environmental impact [33,34,35]. Another important issue presented in the articles [36,37] is the adverse effect of particulate and volatile matter on environmental pollution as well as the deterioration of the friction-mechanical characteristics of the brake. Over longer periods of use, there is a decline in brake effectiveness and efficiency. During these tests, the requirements for vibrations and noise generated by the braking systems described in work [22] are additionally checked. Articles [38,39,40] also pointed out the dependence of the weight wear of friction pads on the vibration-acoustic signal generated by a disc brake. All these issues require a thorough knowledge of the friction and wear process described by various physical models for different pad materials or friction pads [41,42] verified by tests and statistical analyses [28]. Other researchers in works [36,43,44] have presented results from FEM numerical simulations of brake friction pads for various friction materials using Archard and Euler wear equations. In the field of rail vehicle brakes, friction material wear is particularly important from an economic point of view. For example, in vehicles such as electric locomotives or traction units, it is possible to reduce the wear of brake friction material due to the dependence of the braking system on the drive train of these vehicles. In the case of the aforementioned vehicles, the majority of the braking force is provided by electrodynamic (frictionless) braking using traction motors in alternating current operation, which generates additional resistance. Only the missing braking force in the final phase is supplemented by the friction brake because of the traction motor characteristics. At that time, the ratio of the use of an electrodynamic brake during braking versus a conventional friction brake is about 85/15%. Therefore, many rail vehicle research institutes [45,46] are constantly conducting development work on improving electrodynamic brakes and improving their braking performance. This work and research is mainly concerned with ways to store the energy from the operation of the braking system in supercapacitors for longer periods of time [47], the construction of wind turbines on railroad tracks to power railroad lines (traction) [48], and the modification of train schedules to reduce the cases of brake use or to increase the efficiency of brake energy recovery into the overhead line (recuperation phenomenon) [49]. As a result, dust and gas emissions from the friction brake are generated to a minimum, which very significantly reduces the weight wear of friction pads of a pneumatic or electropneumatic brake. However, it should be noted that there are cases during braking when the electro-dynamic brake will be completely disabled. Braking will take place using the air brake (control) and friction brake (generation of braking force). This is the case of emergency (sudden) braking implemented by a passenger or driver.

The main goal of the study was to evaluate the relationship between the weight wear of friction pads and the quantities characterizing the braking process. Based on the results from the tests, the weight wear of particulate friction material from one single braking was determined in grams, at a given braking speed, brake system pressure, mass to be braked, and other quantities. The results were the basis for modeling the weight wear of friction pads, which in the form of particulate matter pollutes the environment. The friction pad weight wear model proposed in the article can be used for both brake system designers and railroad operators. On the basis of the known route of the train, driving parameters, and load on the railroad line, it is possible to calculate the wear of friction material by the brake, for example, an electric multiple unit.

## 2. Methodology and Research Object

**Figure 1:**
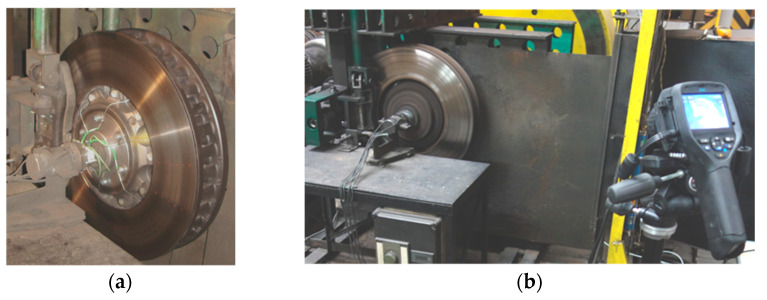

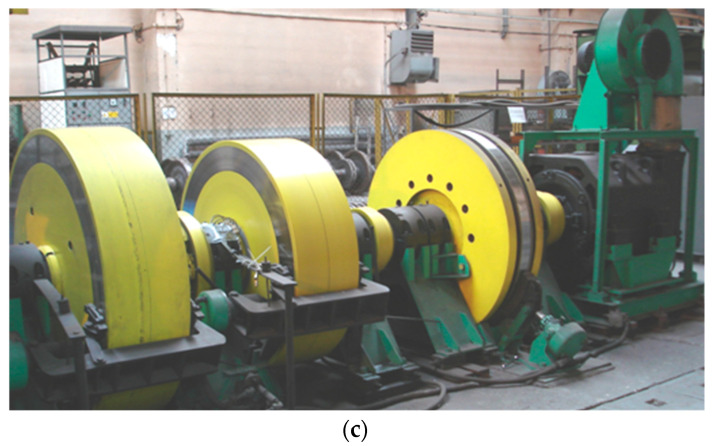
Railroad disc brake testing stand: (**a**) view to the working part of the brake stand with rotating masses, (**b**) view to the tested brake disc with thermovision measurement, (**c**) view of the stand.

The friction pad weight wear test was carried out using a certified inertia dynamometer at the Łukasiewicz Poznan Institute of Technology (Figure 1). The stand allows for certified and homologated tests of friction-mechanical brakes of all rail vehicles. These tests allow for the simulation of all types of braking that can occur in real conditions.

**Figure 2:**
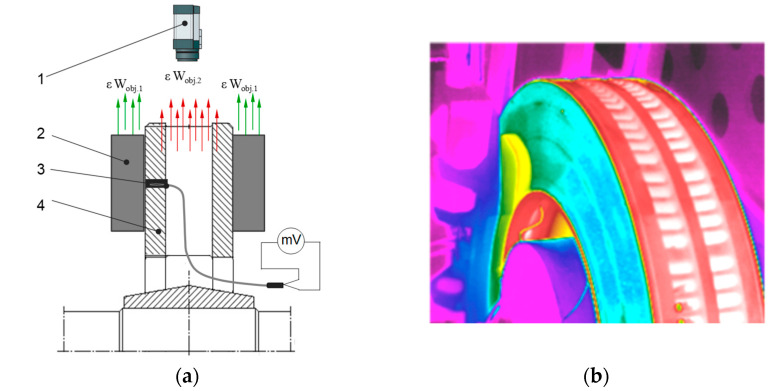
Diagram of thermal imaging test by calorimetric method (**a**), example thermal imaging photo of brake friction pair (**b**); 1—IR camera, 2—friction pad, 3—thermocouple type TP-213K-a-200-200, 4—brake disc, ε—emissivity coefficient, W_obj.1,2_—radiation acting from object 1 (friction pad) 2 (brake disc) on the detector of the thermal camera.

In order to observe, after each braking, the contact of the friction pads with respect to the brake disc, a Flir e60 thermal imaging camera (Teledyne FLIR LLC, Wilsonville, OR, USA) was used in the tests. The thermal imaging tests and the configuration of the thermal imaging camera were carried out in accordance with the work [50]. In the thermal imaging study, the calorimetric method was used to determine the emissivity. In this method, an additional contact meter (thermocouple TP—213K—a200—200 CZAKI THERMO-PRODUCT, Raszyn-Rybie, Poland) was used to measure the temperature of the object (friction pads). Then, the emissivity was changed in the camera settings for so long as to obtain the same temperature value that was obtained earlier with the contact measurement. The same temperature values were obtained at the emissivity of ε = 0.95. Figure 2 shows a schematic of a thermal imaging camera test.

The research was an active experiment based on [51,52]. During the bench tests, various input parameters such as braking speeds and others were intentionally introduced and their effect on the output, i.e., weight wear of friction pads, was recorded. The tests covered organic pads cooperating with ventilated brake disc (KOVIS—Livarna, Štore, Slovenia) types 590 × 110 and 640 × 110 (disc diameter and width in mm) made of grey cast iron EN-GJL-250 (GG-25). The chemical compositions as a percentage of additives are presented in Table 1. The discs were not subjected to thermo-chemical treatment.

**Figure 3:**
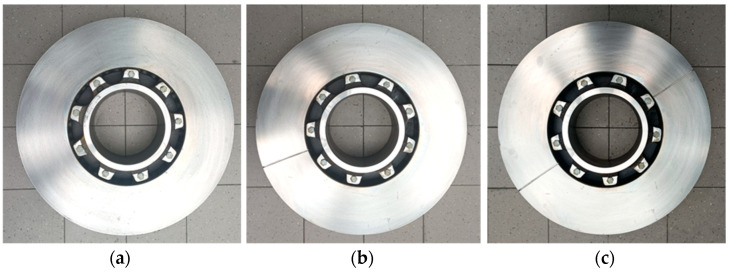
View of the brake discs used during testing: (**a**) 610 × 110 type disc without notch, (**b**) 610 × 110 type disc with one notch, (**c**) 610 × 110 type disc with two notches.

In terms of brake discs, additionally, two transverse notched brake discs were used. This is related to testing the effect of using split brake discs on friction pad wear. One transverse notch was made in one disc, while two notches were made in the other disc, as shown in Figure 3. The notches were made at a depth of 4 mm and their width was 3 mm. The notch dimensions result from the segmented discs used in railroad technology for easy and quick disc replacement without removing the wheels from the axle. The research presented in this paper will also provide information about the influence of the notches in segmented discs on pad wear.

**Figure 4:**
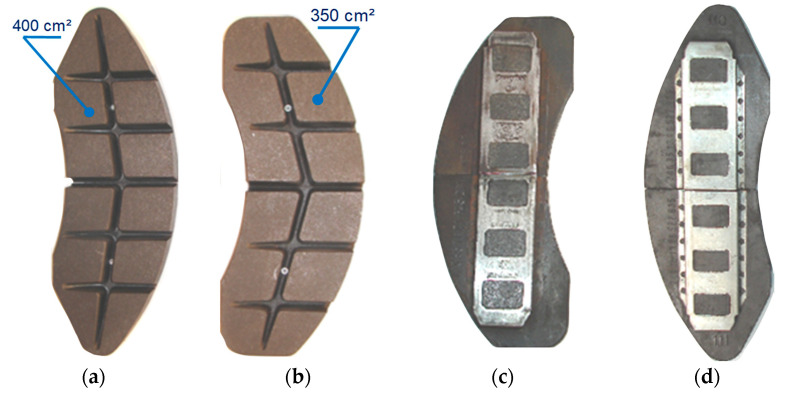
Friction pads used during the test: (**a**) type 350 view from disc contact side, (**b**) type 400 view from disc contact side, (**c**) type 350 view from clamping side with holder, (**d**) type 400 view from clamping side with holder.

Figure 4a,b show a view of the pads used in this study. An important difference in the pads is their working area resulting from the marking, i.e., 350 or 400. This is the area in cm^2^ of the right and left pair of friction pads located on the two sides of the brake disc. On the other side of the pads 3c and 3d, the method of attachment to the holder is presented.

One set of pad consists of four pieces. Tests were carried out on organic (plastic) pads of the FR20H.2 type (FRIMATRAIL Frenoplast, Wołomin, Poland). According to [7], the pads were made of thermosetting resin, organic fibers, shredded metal powders, synthetic elastomer, and friction modifiers. Friction pads are completely asbestos-free compressed friction material from the plastic group (organic material). This type of pad, according to the manufacturer’s declaration, is suitable for brake operation with a specific pressure of up to 70 N/cm^2^, a permissible continuous temperature of up to 375 °C, while the instantaneous temperature cannot exceed 450 °C. During the tests, three sets of each type of pad (400 and 350) were used with different thicknesses (initial linear wear). The first set was new (thickness of 3.5 cm), the second set was worn to a thickness of 2.5 cm, and the third set was worn to a thickness of 1.5 cm.

The bench testing was carried out in compliance with the UIC 541-3 standard with regard to selection of the friction pad pressure to the brake disc and the braking mass per one brake disc. The parameters changed during the friction pad wear tests were the type of brake disc surface (smooth, with one notch and two notches), the thickness of the friction pad as initial wear (new—35 mm, worn to 25 mm and worn to 15 mm), the speed of the onset of braking (v = 50, 80, 120, 160, and 200 km/h), the pressure of the pad to the disc (N = 16, 25, 26, 28, 36, 40, and 44 kN), and the mass to be braked per disc (M_B_ = 4.4, 4.7, 5.7, 6.7, and 7.5 t).

Prior to the start of the main tests, a series of friction lapping brakes were performed. According to [7,54], pre-braking is carried out until lapping of the pads exceeds 75% of the pre-lapping area. After each braking, the pads were removed from the lever system, cleaned with compressed air, and weighed on an electronic balance with an accuracy of 1 gram (electronic balance PR II 15 B CAS, Poznan, Poland). In total, 210 braking tests were performed on the brake bench with different combinations of input parameters. These braking tests do not take into account the lapping process of the pads (10 braking tests for each set, 6 sets give a total of 60 lapping braking tests).

## 3. Analysis of Bench Test Results

The first stage of the research was to determine the increment of weight wear of friction pads as a function of braking speed at different values of pad pressure to the disc and pad thickness. Selected results from measurements of friction pad wear from braking with an applied load of 36 kN and a mass to be braked of 5.7 t, measured after a single braking application, are presented in Table 2.

On the basis of the results in Table 1, it can be seen that as the braking velocity, the number of notches in the disc (its perforation), and the initial wear of the friction pads increase, the weight-related wear of the friction pads increases.

**Figure 5:**
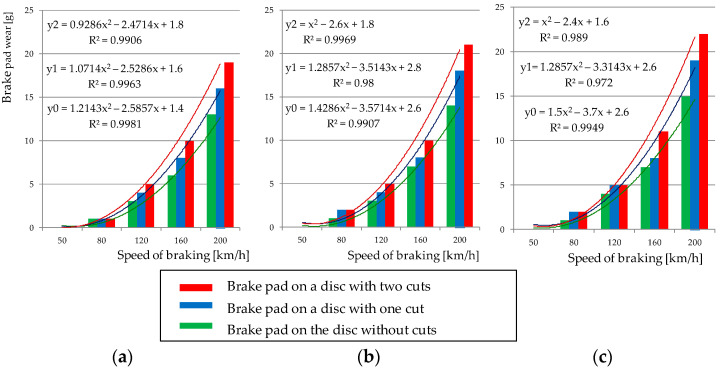
Dependence of friction pad wear as a function of velocity, for braking with load N = 36 kN, braking mass M_B_ = 6.7 t, braking on pads: (**a**) new, thickness 35 mm, (**b**) worn to the thickness of 25 mm, (**c**) worn to the thickness of 15 mm.

Figure 5 shows a graphical representation of the relation between brake pad wear and braking speed for three surfaces of a brake disc (smooth disc without perforations and with perforations in the form of one and two notches) after one braking application. The graphs additionally show approximating quadratic functions for which the highest determination coefficient R^2^ was obtained in relation to the power function and the exponential function. Analyzing Figure 4, it is found that regardless of the pressure of the pad to the disc, the mass to be braked, and the initial linear wear (thickness of the pad), the weight wear can be modeled using a quadratic regression function. The highest coefficient of determination (0.97–0.99) was obtained for this function (regression model) relative to the other regression functions.

**Figure 6:**
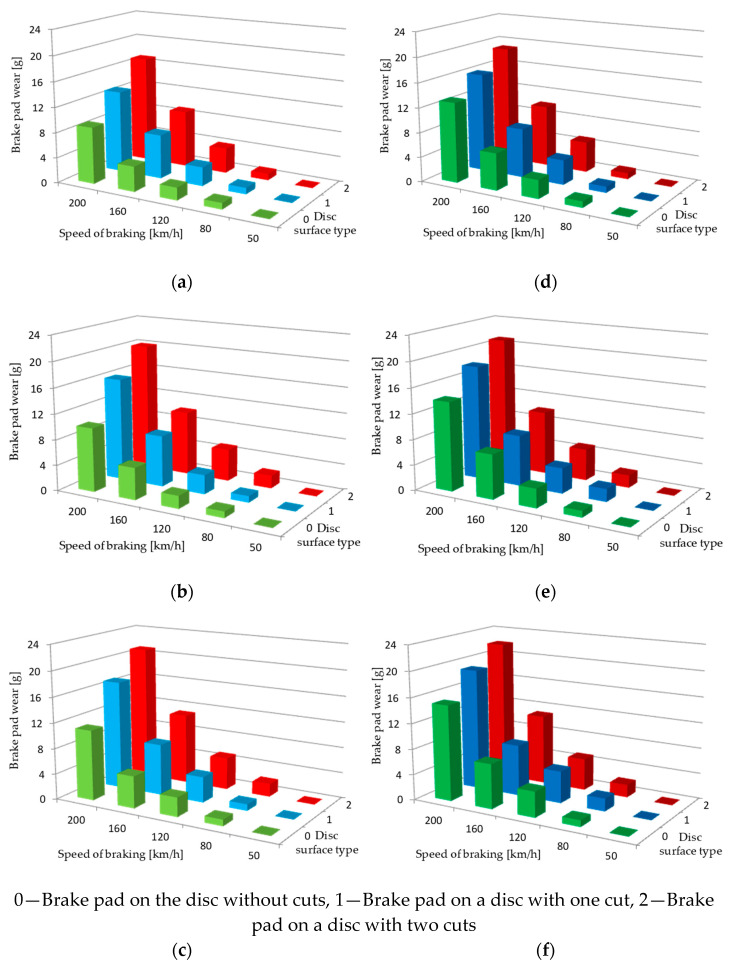
View of weight wear of friction pads under braking: (**a**) N = 25 kN, M_B_ = 5.7 t (for 35 mm pad), (**b**) N = 25 kN, M_B_ = 5.7 t (for 25 mm pad), (**c**) N = 25 kN, M_B_ = 5.7 t (for 15 mm pad), (**d**) N = 36 kN, M_B_ = 5.7 t (for 35 mm pad), (**e**) N = 36 kN, M_B_ = 5. 7 t (for 25 mm facing), (**f**) N = 36 kN, M_B_ = 5.7 t (for 15 mm facing), 0—smooth disc without perforations, 1—disc with one cut, 2—disc with two cut.

Figure 6 shows, for several braking cases (constant pressure and mass to be braked), the dependence of the weight wear of friction pads on three variables, i.e., braking speed and type of disc surface (for a smooth disc and a notched perforated disc).

The bench test was carried out on 210 braking tests at various combinations of speed, pressure, mass to be braked, pad thickness, and the type of brake disc surface proved the existence of the dependence of the weight wear of pads on the input (set) parameters of the braking process.

## 4. Modeling of Weight Wear of Friction Pads

**Figure 7:**
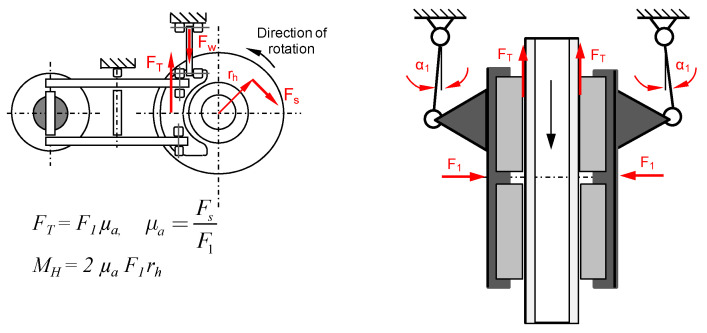
Physical model of friction in a disc brake, F1—forces acting on the brake pad holder, F_s_—tangential force related to the braking radius r_h_, F_T_—frictional forces, α_1_—angle of inclination of the vertical lever during braking, F_W_—force of inertia on the circumference of the wheel during, µa—coefficient of friction at rest, M_H_—braking torque.

On the basis of the results of weight wear tests of friction pads, an attempt was made to model the pad wear on the basis of the following input parameters: type of the disc surface (perforation), contact area of the pads with the disc, initial linear wear of pads, pressure of the pad to the brake disc, braking mass per disc, and speed of the beginning of braking. Figure 7 graphically depicts the physical model of friction (braking process) in a disc brake.

A multiple regression model, also called polynomial regression, was used to model weight wear. In this method, the value of the random variable Y depends on the *i*-th independent characteristic (X_1_, X_2_, … , X_i_). Based on a given sample of test results according to [54], the invariant parameters δ_0_, δ_1_, … δ_k_ used the method of least squares. The following relation was proposed to determine the weight wear of the pad.

(1) $$w_{w}=\delta_{1}D+\delta_{2}S_{P}+\delta_{3}T_{P}+\delta_{4}N+\delta_{5}M_{B}+\delta_{6}v+\delta_{7}v^{2}+\delta_{0}\;\;\; [g]$$

where:

*D*—brake disc surface (0—smooth without perforations, 1—with one notch, 2—with two notches),

*S_P_*—contact surface of pad and disc (2 × 350 cm^2^, 2 × 400 cm^2^),

*T_P_*—thickness of friction pads (new *T_P_*_1_ = 35 mm, worn to *T_P_*_2_ = 25 mm, and *T_P_*_3_ = 15 mm),

*N*—brake pad pressure to brake disc (*N* = 16, 25, 26, 28, 36, 40, and 44 kN),

*M_B_*—braking mass per disc (*M_B_* = 4.4; 4.7; 5.7; 6.7; 7.5 t),

*v*—speed of beginning of braking (*v* = 50, 80, 120, 160, and 200 km/h).

For the model described by relation (1), the parameters of the multiple regression function were determined from Equation (2) [27].

(2) $$r=\frac{\sum_{i=1}^{n}\left(x_{i}-\bar{x}\right)\left(y_{i}-\bar{y}\right)}{\sqrt{\sum_{i=1}^{n}{\left(x_{i}-\bar{x}\right)}^{2}\sum_{i=1}^{n}{\left(y_{i}-\bar{y}\right)}^{2}}}$$

where:

*y*, *x*—the mean values of *x* and *y*,

*y_i_*, *x_i_*—descriptive variables.

The empirical model described by relation (3) was verified and the significance of the regression coefficient system, as well as the significance of individual regression coefficients, were checked. Verification of the empirical models described by relations (3) and (4) was performed. For the weight wear model, the statistical hypothesis for the significance of the regression coefficient system was formulated as follows:

(3) $$H_{o}\;:\;\sum_{k\;=\;\;0}^{n}\delta_{k}^{2}=0;\;\left(k=0,\;1,\;2,\;3,\ldots ,\;7\right)$$

(4) $$H_{1}\;:\;\sum_{k\;=\;0}^{n}\delta_{k}^{2}\neq 0;\;\left(k=0,\;1,\;2,\;3,\ldots ,\;7\right)$$

Rejection of the H_o_ hypothesis means that there are statistical grounds for assuming that there is a linear relationship between the dependent variable and at least one explanatory variable. Snedecor’s F distribution was used in the significance test of the regression model.

To determine the significance of the individual regression coefficients, hypotheses written are as follows:

(5) $$H_{o}\;:\;\delta_{k}=0$$

(6) $$H_{1}\;:\;\delta_{k}\neq 0$$

Student’s t-distribution was used to test hypotheses regarding the significance of all regression coefficients. If the significance of F is less than the assumed significance level α, i.e., α = 0.05, then there are grounds for rejecting the null hypothesis and assuming that there is a linear relationship between the explanatory variable and all explanatory variables included in the models.

For the friction pad weight wear model *w_w_* after statistical testing, Table 3 gives the values of the coefficients (*δ*_0_, *δ*_1_, *δ*_2_, …, *δ*_7_) of the multiple regression function along with the coefficient of determination R^2^.

Analyzing the results of the statistical test in Table 3, it is found that some coefficients, i.e., *δ*_0_ and *δ*_2_ of the *w_w_* model described by relation (1), do not meet the assumed significance level of α < 0.05. These coefficients were removed, and the polynomial regression was determined without taking into account the variable related to the contact area of the pad with the disc (S_P_). Table 4 shows the new statistical test results for the friction pad weight wear model after revising its coefficients.

After verifying the parameters of the multivariate regression model *w_w_*, the final form of the friction pad weight wear model based on the given quantities describing the braking process is as follows:

(7) $$w_{w}=1.78\cdot D-4.14\cdot 10^{-2}T_{P}+8.36\cdot 10^{-2}N+1.17M_{B}-5.92\cdot 10^{-2}v+5.78\cdot 10^{-4}v^{2}-7.35\;\;\; [g]$$

**Figure 8:**
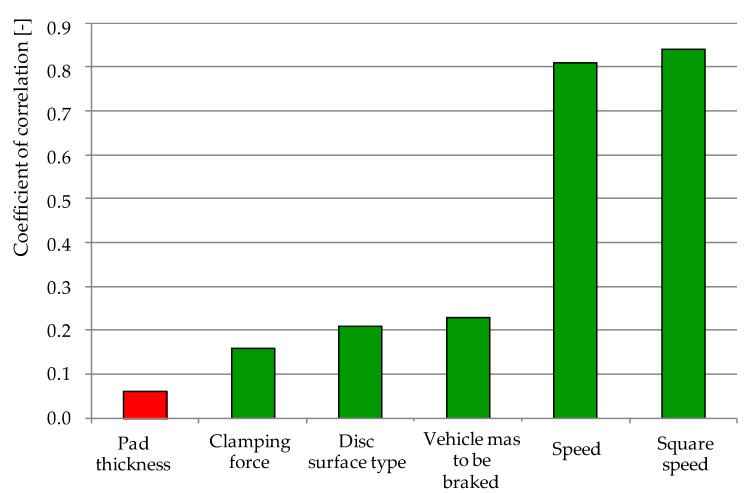
Distribution of the correlation coefficients of the variables of the friction pad weight wear model, green means that as the value of the variable increases, the wear of the pads increases, and red means that as the value of the variable increases, the wear decreases.

In the next step of experiment, the Pearson’s linear correlation coefficient (Table 5) was checked for the analyzed variables, i.e., disc surface type, pad thickness, pad pressure to disc, mass to braking, and braking velocity after verifying the coefficients of the friction pad wear weight model. Figure 8 shows the distribution of the correlation coefficients of the individual variables of the friction pad wear weight model, from the lowest to the highest coefficient value.

On the basis of the values of the correlation coefficient in Table 5, it can be seen that the changes in the value of the weight wear of friction pads are most strongly influenced by the speed of the beginning of braking (r = 0.81). This shows the very strong dependence of *w_w_* on *v*. On the other hand, the type of friction surface of a disc with or without perforations and the mass to be decelerated have a weak effect on the changes in *w_w_*. The correlation coefficient for these variables is in the range of 0.21–0.23. Other variables such as pad pressure to the disc or initial pad wear (thickness) have a low effect on the results of the weight model of friction pad wear.

## 5. Verification of the Friction Pad Weight Wear Model

Then, according to relation (8), the fit of the weight wear model to the test results was verified based on the determination of the relative percentage error [54].

(8) $$\delta =\frac{\left|w-w_{w}\right|}{w}\cdot 100\%$$

where:

*w* weighted wear value from brake bench tests,

*w_w_* value of weight wear determined from the multiple regression model (relation (7)).

Due to the large number of samples *n* > 30, the number of classes *k* was determined based on inequality (9) so that it is possible to determine the distribution of relative error w expressed in percentages [55].

(9) k≤5lnn

**Figure 9:**
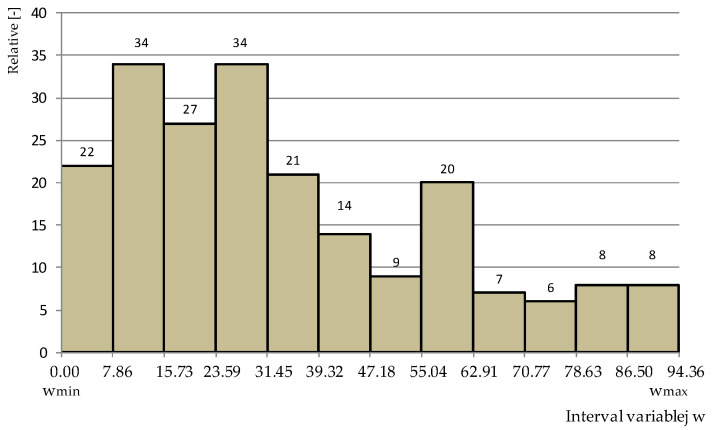
Histogram of relative error counts of the percentage fit of the multiple regression model of friction pad weight wear to test results.

For applications that relation (9) is allowed to determine, the number of classes *k* equals 12. From the relative error data, the maximum value of the variable, w_max_ = 86.50, and the minimum value, w_min_ = 0.0, were determined. Figure 9 shows a histogram of the relative error percentage counts for the 12 classes.

Considering the histogram shown in Figure 9, it is found that the largest number is the relative error due to the mismatch between the multiple regression model and the results from the tests in the range of 7.8–15.7% and 23.6–31.4%, which occurred in 34 cases out of 210 observations. In 22 cases, the error was 0%, which represents 10% of the total results obtained from the model. In 8 cases, the error in fitting the model to the results from the study was 78.6–86.5% and 86.5–94.4%. The average statistical relative error was 28% for all braking.

**Figure 10:**
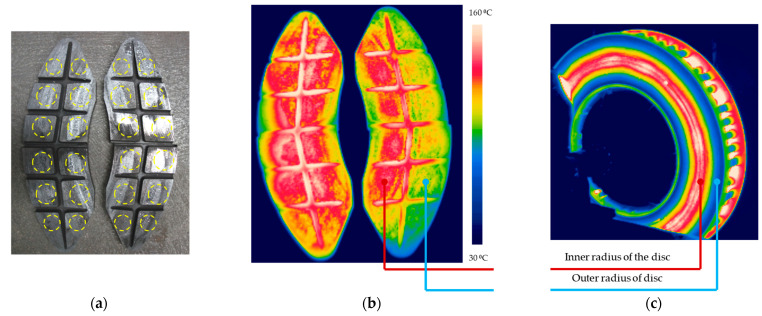
View of the friction pair after testing: (**a**) friction pad view, (**b**) friction pad thermal image, (**c**) brake disc thermal image after braking from v = 200 km/h.

The research on determining the weight wear of friction pads after one braking and its modeling refers to different braking cases for all four friction pads. They constitute the friction pair for one brake disc. The results from model (7) may indicate that the wear is distributed uniformly over all four pads. The carried-out thermal imaging test in parallel with the friction—mechanical tests proved that the contact of the friction pads with the brake disc was uneven, as shown in Figure 10. This was found by removing all friction pads from the brake lever mechanism immediately after braking. The results of these tests are included in Table 6. From each area in the form of a pad circle as in Figure 10a, the average pad temperature was determined. The transverse and vertical expansion grooves were not included in the thermal imaging studies because they are not involved in the friction process.

The thermal imaging tests proved that, in all friction elements, the pad temperature is not the same. The maximum temperature in one of the pad areas was 146.8 °C on the upper left friction pad, while the lowest pad-to-disc contact temperature was 111.3 °C on the lower right pad. In the paper [56], the issue of uneven distribution of pad pressures relative to the disc was explained by the uneven weight distribution of the right and left sides of the brake lever system as well as by the change in geometric dimensions of the brake lever during braking and gradual wear of the pads. The thermal imaging tests showed that the non-uniform temperature distribution on the right brake pads in terms of the inner and outer braking radius affected the variable temperature distribution on the right side of the brake disc. In Figure 10c, it was possible to observe a hot band on the inner radius with the beginning of the formation of hot spots type areas after one braking from 200 km/h.

## 6. Conclusions

This article presents the test methodology and results from the study of weight wear of friction pads on a certified brake testing bench for disc brakes of rail vehicles. On the basis of friction-mechanical tests, 210 braking were performed with various combinations of pressure, speed, mass to be braked and the type of brake disc surface. As a result, the regression model of friction pad wear expressed in grams after each braking was determined.

Based on the testing and modeling of friction pad wear, it was found:

- from the braking process parameters, the significant increase in the weight wear of friction pads is most strongly influenced by braking velocity and the correlation coefficient of the regression model for this parameter was 0.81. For the other input data, the following correlation coefficients were obtained: braking mass r = 0.23 and type of disc surface perforation r = 0.21.
- the pressure of the friction pad to the brake disc and the thickness of the friction pad do not show any influence on the results of the wear weight model (correlation coefficient r below 0.2).
- Thermal imaging tests proved about uneven temperature distribution on the pads on the right and left sides. This indicates uneven pressure distribution and uneven wear of the pads located on the two sides of the brake disc.
- The weight wear model proposed in the article applies to all pads, regardless of their side of contact with the brake disc.
- despite the symmetrical lever system, the pads in a disc brake do not wear uniformly, which was proven by thermographic imaging tests.

In further work, it is planned to include other variables from the group of braking process parameters for modelling wear of friction pads, such as changing temperatures of brake discs and the impact of environmental factors, e.g., rainfall and the related phenomenon of aquaplaning.

## Figures and Tables

**Table 1:** Chemical composition in % of elements for cast iron discs used during the tests and mechanical properties [53].

Chemical Composition							
C	Si	Mn	P	Cu	S	Ce	Sc
3.5	1.5	0.7	0.5	0.15	-	-	-
**Mechanical properties**							
Modulus of elasticity E [N/mm^2^]	Thermal conductivity coefficient λ [W/mK]	Heat capacity (specific heat) Cw [J/kgK]	Thermal expansion coefficient α [(1/K)·10^−5^]	Elongation A5 [%]	Density ρ [(kg/m^3^)·10^3^]	Tensile strength Rm [N/mm^2^]	Hardness HB
110,000	50.2	598.7	1.80	0.5	7.1	250	180

**Table 2:** Friction pad wear weight in grams, results after braking with N = 36 kN and M_B_ = 5.7 t.

Velocity km/h	Disc without Notch			Disc with One Notch			Dial with Two Notches		
	Brake Pad Thickness T_P1_ = 35 mm	Brake Pad Thickness T_P2_ = 25 mm	Brake Pad Thickness T_P3_ = 15 mm	Brake Pad Thickness T_P1_ = 35 mm	Brake Pad Thickness T_P2_ = 25 mm	Brake Pad Thickness T_P3_ = 15 mm	Brake Pad Thickness T_P1_ = 35 mm	Brake Pad Thickness T_P2_ = 25 mm	Brake Pad Thickness T_P3_ = 15 mm
50	0	0	0	0	0	0	0	0	0
80	1	1	1	1	2	2	1	2	2
120	3	3	4	4	4	5	5	5	5
160	6	7	7	8	8	8	10	10	11
200	13	14	15	16	18	19	19	21	21

**Table 3:** Summary of statistical test results for the disc brake friction pad wear model.

Coefficient	Value	Value F *
*δ*_1_	1.53	4.41∙10^−7^
*δ*_2_	−2.17∙10^−2^	0.21
*δ*_3_	−4.14∙10^−2^	3.23∙10^−2^
*δ*_4_	8.48∙10^−2^	5.98∙10^−5^
*δ*_5_	1.18	1.46∙10^−11^
*δ*_6_	−5.92∙10^−2^	7.03∙10^−4^
*δ*_7_	5.78∙10^−4^	4.01∙10^−15^
*δ*_0_	−3.21	0.37
*R*_2_	0.84	
*F* **	2.00∙10^−77^	

* significance for individual regression coefficients. ** system wide relevance.

**Table 4:** Summary of statistical test results for the disc brake friction pad wear model after verification of model coefficients.

Coefficient	Value	Value F *
*δ*_1_	1.78	2.04∙10^−14^
*δ*_2_	−4.14∙10^−2^	3.26∙10^−2^
*δ*_3_	8.36∙10^−2^	7.56∙10^−5^
*δ*_4_	1.17	1.87∙10^−11^
*δ*_5_	−5.92∙10^−2^	7.14∙10^−4^
*δ*_6_	5.78∙10^−4^	4.22∙10^−15^
*δ*_0_	−7.35	1.03∙10^−6^
*R*_2_	0.84	
*F* **	3.12∙10^−78^	

* significance for individual regression coefficients. ** system wide relevance.

**Table 5:** Correlation matrix.

Variable	Disc Type *D*	Pad Thickness *T_P_*	Pressure *N*	Mass *M_B_*	Velocity *v*	Velocity *v*^2^	Correlation Coefficient
Disc type *D*	1	−1.42·10^−18^	−0.05	−0.04	5.17·10^−18^	5.17·10^−18^	0.21
Pad thickness *T_P_*	−1.42·10^−18^	1	9.41·10^−18^	−4.1710^−18^	0	0	−0.06
Pressure *N*	−0.05	9.41·10^−18^	1	0.28	5.71·10^−18^	5.32·10^−17^	0.16
Mass *M_B_*	−0.04	−4.1710^−18^	0.28	1	1.52·10^−17^	2.56·10^−18^	0.23
Velocity *v*	5.17·10^−18^	0	5.71·10^−18^	1.52·10^−17^	1	0.98	0.81
Velocity *v*^2^	5.17·10^−18^	0	5.32·10^−17^	2.56·10^−18^	0.98	1	0.84
Correlation coefficient	0.21	−0.06	0.16	0.23	0.81	0.84	1.0

**Table 6:** Temperature distribution results on friction pads.

Left Upper Pad			Right Upper Pad		
Measurement	Outer Radius of Pad	Inner Radius of Pad	Measurement	Outer Radius of Pad	Inner Radius of Pad
1	137.2 °C	146.8 °C	1	134.9 °C	120.8 °C
2	139.0 °C	141.4 °C	2	140.3 °C	124.5 °C
3	139.7 °C	138.2 °C	3	135.4 °C	123.3 °C
**Bottom left pad**			**Bottom right pad**		
**Measurement**	**Outer radius of pad**	**Inner radius of pad**	**Measurement**	**Outer radius of pad**	**Inner radius of pad**
1	146.5 °C	139.7 °C	1	139.7 °C	124.5 °C
2	142.1 °C	141.7 °C	2	139.9 °C	119.3 °C
3	134.3 °C	136.1 °C	3	124.8 °C	111.3 °C
Mean value	139.8 °C	140.7 °C	Mean value	135.8 °C	120.6 °C
Difference ^1^	0.9 °C		Difference ^2^	15.2 °C	
Difference ^3^	20 °C				

^1^ temperature difference between the inner and outer radius of the braking on the left pad, ^2^ difference in temperature between the inner and outer braking radius on the right pad, ^3^ difference between the highest and the lowest mean temperature on all pad surfaces.

## Data Availability

The data presented in this study are available on request from the corresponding author.
